# Fabrication of Large Area Fishnet Optical Metamaterial Structures Operational at Near-IR Wavelengths

**DOI:** 10.3390/ma3125283

**Published:** 2010-12-15

**Authors:** Neilanjan Dutta, Iftekhar O. Mirza, Shouyuan Shi, Dennis W. Prather

**Affiliations:** Department of Electrical and Computer Engineering, University of Delaware, Newark, DE 19716, USA; E-Mails: iomirza@udel.edu (I.O.M.); sshi@udel.edu (S.S.); dprather@ee.udel.edu (D.W.P.)

**Keywords:** fishnet metamaterial, deep UV lithography, negative refraction, ‘lift-off’ process

## Abstract

In this paper, we demonstrate a fabrication process for large area (2 mm × 2 mm) fishnet metamaterial structures for near IR wavelengths. This process involves: (a) defining a sacrificial Si template structure onto a quartz wafer using deep-UV lithography and a dry etching process (b) deposition of a stack of Au-SiO_2_-Au layers and (c) a ‘lift-off’ process which removes the sacrificial template structure to yield the fishnet structure. The fabrication steps in this process are compatible with today’s CMOS technology making it eminently well suited for batch fabrication. Also, depending on area of the exposure mask available for patterning the template structure, this fabrication process can potentially lead to optical metamaterials spanning across wafer-size areas.

## 1. Introduction

After Veselago put forth the idea of a left-handed medium [1], where negative permittivity and permeability values could in fact lead to a negative refractive index, a lot of research has been conducted to realize metamaterials which exhibit negative refraction at various frequency ranges [2,3,4]. In recent years optical metamaterials of various configurations have garnered a lot of attention. Among these, the ‘fishnet’ metamaterial structure has been one of the more extensively explored optical metamaterial structures [5,6,7,8].

**Figure 1:**
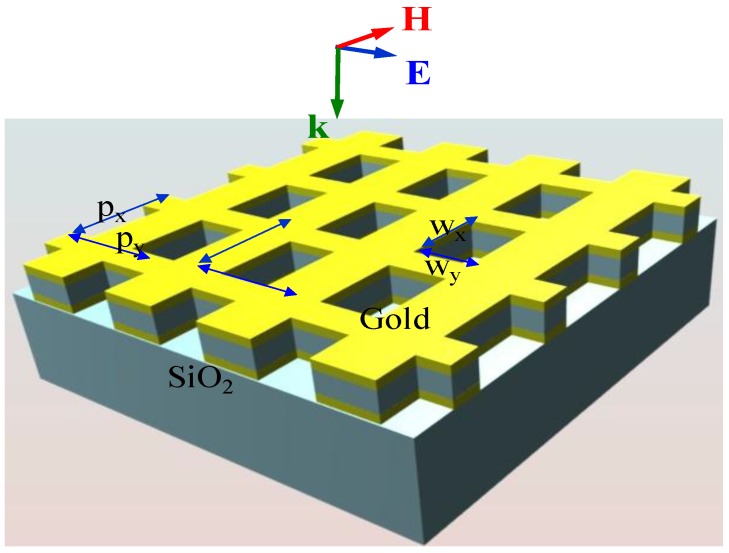
A schematic of a fishnet structure.

The fishnet metamaterial structure comprises a pair of thin metallic layers, with periodically arrayed rectangular perforations or ‘holes’, separated by a thin dielectric filler layer, as illustrated in Figure 1. The dimensions and periodicities of these holes along with the materials constituting the fishnet structure have been varied to bring about left-handed devices in mid-IR and near-IR wavelengths [5,6,7]. Recent studies have also demonstrated fishnet structures with multiple stacks of metal-dielectric-metal layers which have been shown to enhance the left-handed behavior of these devices and allows for direct demonstration of these properties [8].

For an incident optical signal polarized as seen in Figure 1, the metal-dielectric-metal wires lying parallel to the magnetic field form a LC circuit whereby anti-parallel currents are induced in the metal layers straddling the dielectric layers. This gives rise to a magnetic field which counteracts that of the incoming optical signal [5,6,7,8]. This results in a negative permeability which is a consequence of the magnetic resonance. On the other hand, the wires lying parallel to the electric field of the incoming optical signal are essentially dilute Drude metals below their effective plasma frequency which bring about a negative permittivity [5,6,7]. For the frequency range where the above described resonant behavior of the electric and magnetic responses coincide, the refractive index (real part) of the media also becomes negative [8,9,10,11,12]. Negative refraction can be used to realize a variety of novel applications, *i.e*., sub-diffraction imaging [13] and cloaking [14], to name a few.

Fishnet structures operational at near-IR wavelengths, comprise features on the order of a few hundred nanometers [5]. This renders conventional *i*-line lithography processes unsuitable for fabricating these devices. Therefore, for the most part, these structures are fabricated using e-beam lithography (EBL) [10] and nano-imprint lithography (NIL) [15]. While EBL can very easily lead to devices with feature sizes on the order of a few tens of nanometers, fabrication of large area devices can be prohibitively expensive and time consuming. NIL can lead to large area metamaterials with nanometer scale features at very high throughputs. However, NIL is not yet fully compatible with contemporary semiconductor processing techniques.

Previously [16], we demonstrated a deep-UV (DUV) lithography [17] based fabrication process for optical metamaterials that was suitable for mass fabrication of optical fishnet metamaterial devices over potentially wafer-size areas. The above involved patterning a sacrificial quartz template structure, followed by deposition of metallic and dielectric layers and finally a buffered oxide etch (BOE) process to ‘lift-off’ the sacrificial template and yield the final structure. However, these structures featured Si as the dielectric spacer layer. This was due to the fact that Si can be deposited by e-beam evaporation, a ‘line of sight’ process which prevents coating of the template sidewalls and hence facilitates the template ‘lift-off’ step. Given that our primary aim was towards developing a fabrication process based on DUV lithography which would bring about mass producible large-area devices, the smallest structure that we could successfully resolve with DUV lithography technique in conjunction with a Si dielectric spacer layer would be operational at high near-IR (2–2.5 µm) wavelengths. The high permittivity value of the Si layer pushes the operational wavelengths towards higher wavelengths.

In this study we have modified and refined the process demonstrated in our previous study and were successful in integrating SiO_2_ as the dielectric spacer. As per our simulation results, the significantly lower permittivity of SiO_2_ has rendered the devices fabricated operational at 1.5 µm. For the devices fabricated in this study we utilized a sacrificial template structure made of Si. The SiO_2_ dielectric layer was deposited using a plasma enhanced chemical vapor deposition (PECVD) tool which is a highly isotropic deposition method which can cover the sidewalls of the Si template structure. Notwithstanding, we were able to successfully carry out a ‘lift-off’ of the sacrificial template structure. We also utilize a plasma etch process [18] in this study to pattern the Si sacrificial template structure which allows for precise control over the side wall angles of the template structures. This greatly enhances the effectiveness of the overall fabrication method. In contrast, our previous study utilized a quartz template structure that had to be patterned using a combination of dry and wet etching steps which allowed for a far lesser degree of control over the template sidewall angles. The devices fabricated in this study are also significantly larger (2 mm × 2 mm) as compared to the area of devices presented in our previous study (100 µm × 100 µm). Furthermore, we address a critical problem of Au aggregation during the template removal stage which was imperceptible in our previous work. The tendency of Au nano-particles to aggregate together and stick onto metallic surfaces [19] can result in the materials removed with the sacrificial template ‘lift-off’ process to redeposit on top of the device structure. This problem was not faced in our previous study due to the fact that the final BOE etch could be carried out within a sonicator, and also partially due to smaller sample sizes. For the process demonstrated in this study, the final template ‘lift-off’ step is accomplished by a KOH wet etch process which has to be maintained at an elevated temperature, which precludes the use of a sonication bath.

The key advantage of the fabrication process lies in the fact that the DUV lithography process used to define the template structure allows for optical metamaterials that can span across very large areas. Furthermore, this fabrication process, unlike EBL and NIL, uses processes which are compatible with existing CMOS technology and hence can be implemented for mass production of these devices.

## 2. Modeling Results

**Figure 2:**
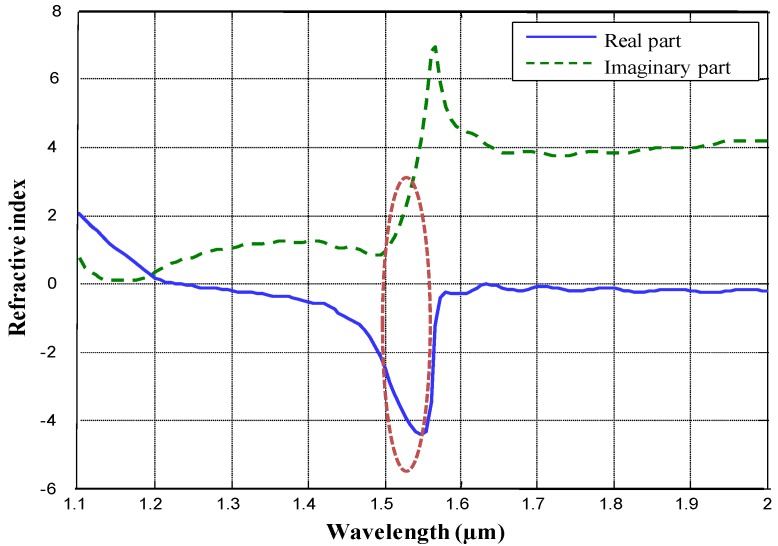
Simulated results for the real and imaginary part of the refractive index depicted by the blue and green curves respectively. The red dotted region highlights the high FOM region.

The design of the fishnet structure was extensively modeled using rigorous Finite Domain Time Difference (FDTD) analysis [20], which takes into account the plasmonic properties of Au. The FDTD simulations provided the reflection and transmission data for the simulated structure which was then used to calculate its refractive index [21,22,23]. The fishnet structure simulated corresponded to the smallest template structure that we could resolve with DUV lithography, which in accordance with the nomenclature established in Figure 1, consisted of rectangles with widths w_x_ = 400 nm and w_y_ = 280 nm in the x and y axes respectively at a periodicity of 650 nm in both axes. We have used Au as the metal sandwiching layers and SiO_2_ as the dielectric spacer material. The metallic layers have a thickness of 30 nm. The oxide layer has a thickness of 60 nm. In Figure 2 we have provided the simulation results for the real and imaginary parts of the refractive index as a function of wavelength.

One can observe the resonant behavior of the structure in terms of the real and imaginary parts of the refractive index around 1.57 μm. Whereas the real part of the refractive index can be seen to be sharply trending negative, the positive imaginary part of the refractive index also increases significantly which indicates enhanced optical losses at resonance. However, at slightly lower than resonant wavelengths, *i.e*., 1.5 μm–1.55 μm (indicated in the red dotted region in Figure 2), the real part of the refractive index approaches −4 and the imaginary part varies between 1.5 and 2. Thus, a figure of merit (FOM), |Re(n)|/Im(n), of 2 and above can be realized with this structure. This is comparable to the FOM figures presented in other studies [24,25].

## 3. Fabrication Process

**Figure 3:**
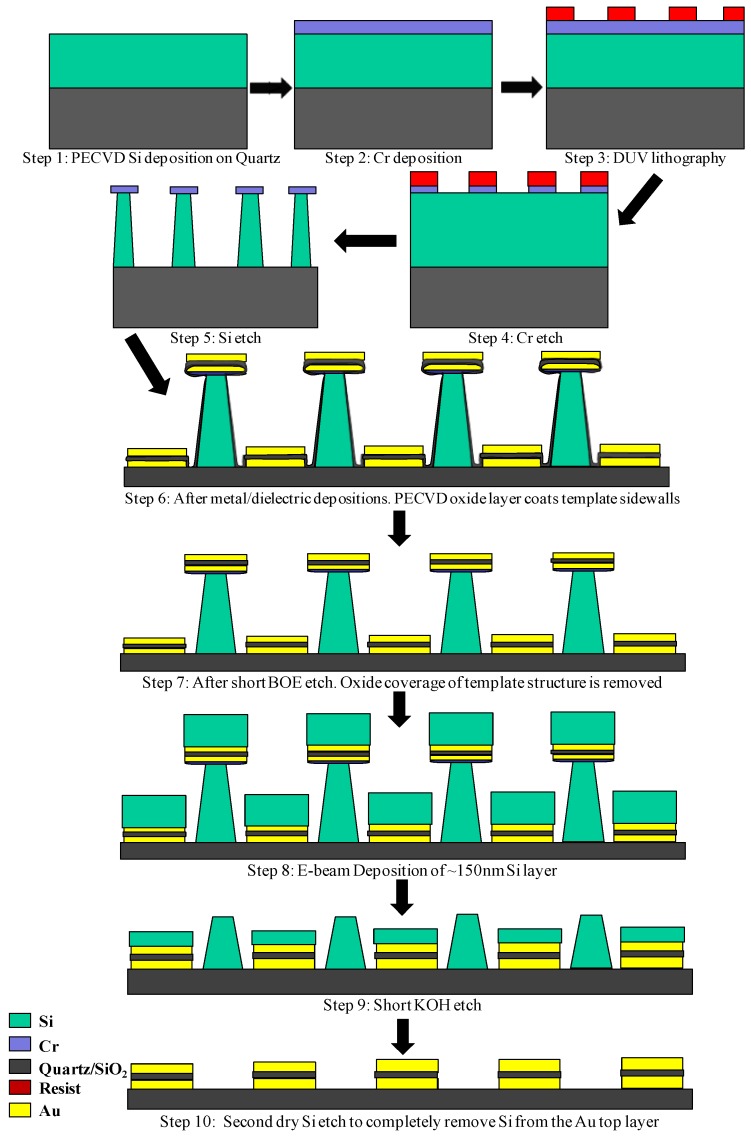
The fabrication process.

Figure 3 outlines the steps for the process developed in this study. This process starts with a deposition of 350 nm of Si on top of a quartz substrate using a PECVD tool followed by an e-beam evaporator deposition of a 50 nm Cr layer, as depicted in Steps 1 and 2 of Figure 3. In Step 3, a thin layer of SU-8 resist (~200 nm) is spin-coated onto the above described substrate and exposed to DUV (220 nm) radiation in a mask aligner using a dark-field exposure mask with rectangular holes corresponding to the fishnet structure as described in the previous section. The mask aligner is fitted with a set of filters to remove long-wavelength components from the UV source. Additionally, a filter with a transmission window of 30 nm around 220 nm is directly placed over the exposure mask with the SU-8 coated sample substrate placed against it in ‘hard’ contact. Elimination of longer UV wavelengths is essential to prevent diffracted radiation from crosslinking the resist layer in undesired areas. Calibrating the UV exposure dose is also necessary since SU-8 resist is highly absorptive at DUV wavelengths. A lower than optimal exposure dose will prevent the exposed patterns from anchoring down to the substrate whereby they will be removed during development. On the other hand, prolonged exposure will lead to some of the diffracted wavelengths exceed the threshold exposure dose resulting in crosslinked resist in undesired areas. After exposure, the samples are reverse baked and developed to yield the complimentary template structure comprising resist islands corresponding to the holes in the fishnet structure.

In Step 4, chlorine chemistry is utilized in an Inductively Coupled Plasma (ICP) tool to etch the Cr layer with the SU-8 pattern developed in Step 3 as the etch mask. This patterned Cr layer forms the etch mask in Step 5 where another dry etch process is used to etch through the underlying PECVD deposited Si layer to form the sacrificial template structure. The sacrificial template corresponds to the holes in the fishnet structure. The ICP etch process used for etching the Si layer comprises alternating etching and passivation steps [18]. The duration of these steps can be tuned to precisely control the anisotropy of the etch process. Thus, the template pillars formed at the conclusion of Step 5 had a negative sidewall angle which is necessary to successfully carry out a ‘lift-off’ process. The Cr etch-mask used for the Si etching step affords etch selectivity of well over 100:1.

After etching the template structure, Au-SiO_2_-Au layers are deposited, as shown in Step 6. An e-beam evaporator has been used for deposition of Au. The oxide layer has been deposited in a PECVD tool. The thicknesses of the Au and the oxide layers are approximately 30 nm and 60 nm respectively, in agreement with our simulation results. 5 nm Cr layers are deposited at the quartz/Au and oxide/Au interfaces to mitigate the poor adhesion properties of Au.

In Step 7, a short BOE etch (~5 seconds) is conducted. This is done to remove oxide coverage on the template sidewalls which can prevent removal of the template structure.

After Step 7, one could proceed to remove the Si template pillar structure with a wet etch process using KOH. However, when we attempted to do so, the entire sample structure would be littered with Au-SiO_2_-Au discs removed from the top of the template structure. This is due to the tendency of Au nano-particles to aggregate and to cling on to metallic surfaces as discussed earlier. This can be avoided to a certain extent by performing the final KOH etch process within a sonication bath. However, the need for maintaining the KOH solution at an elevated temperature can pose problems with regards to this approach. Steps 8 and 9 were incorporated in the fabrication process to address the issue of Au aggregation.

**Figure 4:**
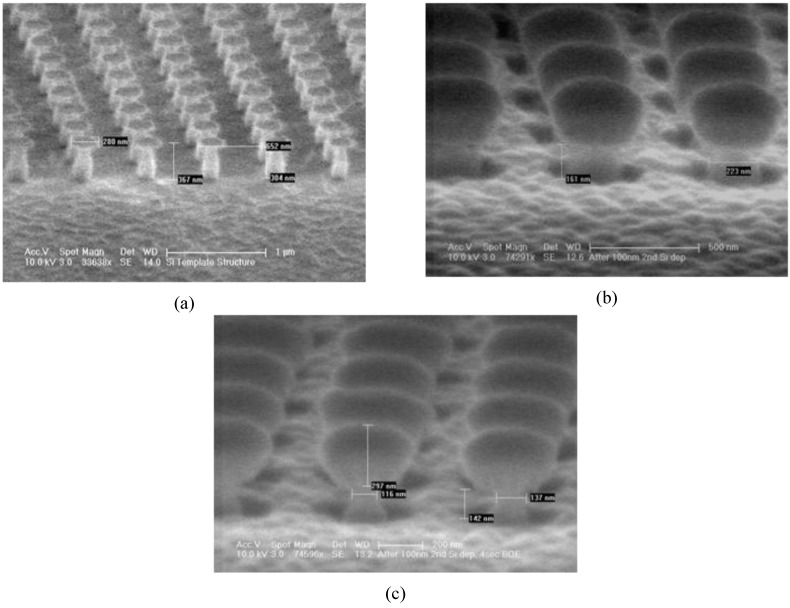
(a) The template pillars after dry etching; (b) Sample after deposition of Au-oxide-Au layers; (c) Sample after ~7seconds BOE etch.

In Step 8, we deposited a thick (~150 nm) Si layer in an e-beam evaporator. The ‘line of sight’ e-beam deposition process ensured that this Si layer did not coat the template sidewalls. The thickness of this Si layer deposited had to be thicker than the width of the Si-template pillars at their narrowest point (~100 nm, near the top of the pillars as depicted in Figure 4(c)). In Step 9 a short (~8 seconds) wet etch with 20% KOH at 60 °C was conducted. This removed a part of the template pillars, along with the Au-SiO_2_-Au layers lying above them, while ensuring that a part of the e-beam deposited Si layer (Step 8, Figure 3) shielded the top-surface of the fishnet structure.

The KOH solution etches the Si template structure at a faster rate than the e-beam deposited Si layer due to the following reasons. Firstly, the isotropic KOH etch process attacked the template structure from every direction as opposed to e-beam deposited Si layer at the base of the templates which could be etched only in a top-down direction. Secondly, the e-beam evaporator deposited Si layer was much denser as compared to the PECVD deposited Si layer used to pattern the sacrificial template structure. Therefore, after the short KOH etch of Step 9 of the fabrication process, a Si layer of ~50 nm survives and covers the top surface of the final structure as shown in Figure 3. This surviving Si layer shields the metallic top-surface of the fishnet structure and significantly reduces Au-SiO_2_-Au discs from re-depositing. Any remaining Au-SiO_2_-Au discs still adhering to the device structure are easily removed by wiping the samples gently with laboratory wipes. The Si layer coating the sample top-surface prevents the devices structures from being scratched when the samples are wiped with laboratory wipes.

The fabrication process is concluded in Step 10 when a dry etching step is used to remove the remaining Si layer from the top of the device structure. The underlying Au layer remains unaffected by the Si dry etch.

## 4. Fabrication Results

Figure 4(a) is an SEM image of a sample after the dry etching step which forms the template structure (Step 5 of Figure 3). The negative side wall angles of the etched ‘pillars’, apparent from Figure 4(a), are beneficial towards implementing the ‘lift-off’ process. Figure 4(b) shows a sample after deposition of the Au-SiO_2_-Au layers (Step 6, Figure 3). One can observe that the widths of the pillars are approximately 200 nm. In comparison, in Figure 4(c), which depicts a sample after the short BOE etch (Step 7 of Figure 2), one can observe that the width of the pillars are only about 100 nm. This indicates a successful removal of the oxide coverage of the Si template.

A more anisotropic or ‘line of sight’ deposition method (*i.e.*, sputtering) for SiO_2_ deposition would have obviated Step 7 of the fabrication process since the template side walls would not be coated during oxide deposition. Deposition of the oxide layer using a sputtering system will also allow for the deposition of multiple stacks of Au-SiO_2_-Au layers which can enhance the left-handed properties of the structure and allow for direct demonstration of the negative refraction properties [8]. We intend to implement such processes in our future work.

**Figure 5:**
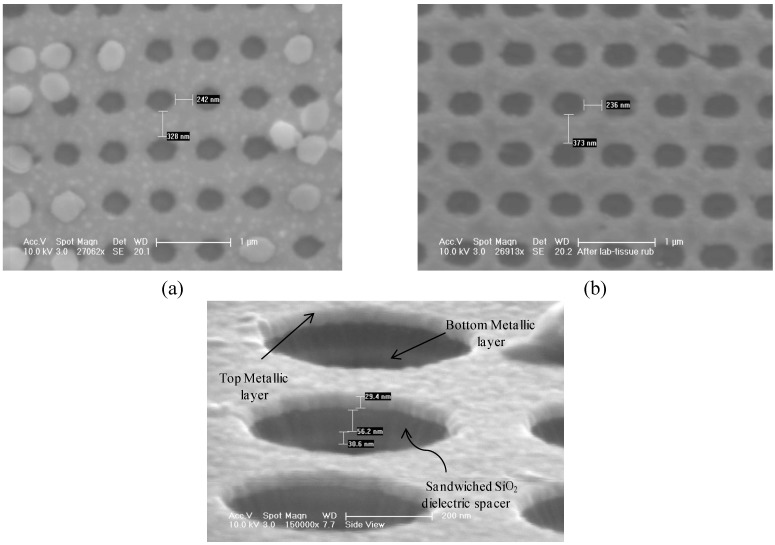
(a) Top-view of a fabricated device with Au-SiO_2_-Au discs re-deposited on to device structure; (b) Top-view of a fabricated device after Step 9 of fabrication process; (c) Side-view of a fabricated device.

Figure 5(a) depicts a sample after the KOH wet etch was used to remove the template structure right after Step 7 of Figure 3. One can observe Au-SiO_2_-Au discs, which were atop the template structure prior to the KOH etching step, adhering to the sample structure. This is due to the tendency of Au nano-particles to aggregate with each other and to cling on to metallic surfaces as we discussed earlier.

In order to avoid the Au aggregation problem, we added Steps 8 and 9 as described in the previous sections. Figure 5(b) depicts a sample after Step 9. One can clearly observe that the sample is devoid of any Au-oxide-Au discs that could be seen in Figure 5(a). A very high degree of uniformity is also apparent. Figure 5(c) depicts a side-view of a sample after the final step of the fabrication process. One can observe the top and the bottom Au metallic layers, each about 30nm thick, with the SiO_2_ dielectric layer, about 60 nm thick, sandwiched in between.

## 5. Conclusions

We have demonstrated a fabrication process for large area metamaterial structures for utilization as a negative index material at 1.5 µm. This process involves lithographically patterning a sacrificial template structure, deposition of thin metallic and dielectric layers and ultimately the removal of the sacrificial template structure to yield fishnet device structure. This fabrication process provides a number of significant advantages. For instance, given that the sacrificial template structure is lithographically patterned, this fabrication process is ideally suited for fabrication of these devices over very large areas. Also, all of the processes used in this fabrication method are compatible with the current semiconductor processing techniques whereby this fabrication process renders these devices mass producible. Currently we are working towards characterizing these devices and considering several structural and dimensional artifacts with regards to the fabricated devices. For instance we will be considering how slight variations of the ‘hole’ dimensions can alter the resonant characteristics of the devices. The above will be the subject of a subsequent publication.
